# Navigating Glioma Complexity: The Role of Abnormal Signaling Pathways in Shaping Future Therapies

**DOI:** 10.3390/biomedicines13030759

**Published:** 2025-03-20

**Authors:** Qiang Chen, Jin Jin, Pian Li, Xiuping Wang, Qianyan Wang

**Affiliations:** 1 Department of Pharmacy, Liyuan Hospital, Tongji Medical College, Huazhong University of Science and Technology, Wuhan 430077, China; tjly15917@163.com; 2 Department of Rehabilitation, Liyuan Hospital, Tongji Medical College, Huazhong University of Science and Technology, Wuhan 430077, China; jinjinxuyu@163.com; 3 Liyuan Cardiovascular Center, Liyuan Hospital, Tongji Medical College, Huazhong University of Science and Technology, Wuhan 430077, China; 2018ly0902@hust.edu.cn

**Keywords:** glioma, signaling pathways, targeted inhibitors, gene therapy, nanomedicine

## Abstract

Gliomas are a type of highly heterogeneous and invasive central nervous system tumor. Traditional treatment methods have limited efficacy, and the prognosis for patients remains poor. Recent studies have revealed the crucial roles of several abnormal signaling pathways in the pathogenesis of gliomas, including the Receptor Tyrosine Kinase/Rat Sarcoma Virus Oncogene/Phosphatidylinositol-3-Kinase (RTK/RAS/PI3K) pathway, the Wingless-Related Integration Site/β-Catenin (Wnt/β-Catenin) pathway, the Hippo/YAP (Hippo/Yes-associated protein) pathway, and the Slit/Robo (Slit Guidance Ligands/Roundabout) signaling pathway. These pathways play extremely vital roles in tumor proliferation, invasion, and treatment resistance. This article comprehensively and systematically reviews the molecular mechanisms of these signaling pathways, deeply summarizing the research progress of various treatment strategies, including targeted inhibitors, gene therapy, and nanomedicine against them. Moreover, the combination of targeted therapy and personalized treatment regimens is expected to overcome the current treatment bottleneck and provide a more favorable survival prognosis for glioblastoma patients.

## 1. Introduction

Gliomas are a type of malignant tumor of the central nervous system, mainly including astrocytomas, oligodendrogliomas, ependymomas, and the most aggressive glioblastoma (GBM) [1]. Considering their high molecular heterogeneity and rapid growth characteristics, the existing standard treatment methods, namely surgery, radiotherapy, and chemotherapy, have restricted overall efficacy, and the five-year survival rate of patients remains at a relatively low level [2]. Recently, with the continuous deepening of the research on glioma biology, the RTK/RAS/PI3K, Wnt/β-Catenin, Hippo/YAP, and Slit/Robo signaling pathways have played a vital role in tumor occurrence, development, and treatment resistance [3,4]. GBM is the most common and aggressive primary brain glioma in adults, with its heterogeneity and treatment resistance showing a close relationship to Wnt/β-Catenin signaling. Glioma stem cells (GSCs) in GBM exhibit high plasticity and play key roles in drug resistance, immune evasion, and tumor recurrence, underscoring the importance of this pathway in GBM research. Therefore, an in-depth understanding of these pathways’ molecular mechanisms and the development of targeted therapeutic strategies is crucial for extending the survival of GBM patients. Furthermore, because GSCs possess drug resistance and self-renewal capacity and play a pivotal role in tumor recurrence and invasion, they have become a central focus in current glioma research [5].

## 2. Key Signaling Pathways in GBM

### 2.1. RTK/RAS/PI3K Pathway

The RTK/RAS/PI3K pathway has a vital impact on the development and progression of gliomas, primarily via the precise regulation of cell proliferation, survival, and metabolism. The activation of this pathway begins with the specific binding of growth factors to receptor tyrosine kinases (RTKs). After binding, RTKs undergo autophosphorylation, which can generate multiple phosphorylation sites. These phosphorylation sites can recruit and activate the guanine nucleotide-binding protein RAS. As a small GTPase, activated RAS acts on downstream PI3K, thus initiating a signaling cascade [6]. PI3K serves as a key signal transducer in this pathway, which can catalyze the conversion of PIP2 to PIP3. Once PIP3 is generated, it further recruits and activates protein kinase B (AKT). Activated AKT exerts extensive biological regulatory functions. Additionally, it can precisely regulate the activity of the mammalian target of rapamycin (mTOR) complex by phosphorylating a series of downstream effector proteins. The change in the activity of the mTOR complex drives cell proliferation, inhibits apoptosis, and promotes metabolic reprogramming to fulfill the requirements of rapid tumor cell growth. In addition, mTOR signaling can further promote the rapid division of tumor cells and the malignant progression of tumors by activating genes related to protein synthesis and cell growth [7].

### 2.2. Wnt/β-Catenin Pathway

In the complex biological process of gliomas, the Wnt signaling pathway exerts an indispensable and critical role. When Wnt ligands specifically bind to Frizzled receptors and low-density lipoprotein receptor-related protein 5/6 (LRP5/6) co-receptors, the “switch” of signal transduction is instantaneously turned on [8]. This binding event activates the downstream Dishevelled (Dvl) protein. The activated Dvl protein acts on the complex composed of Axin, adenomatous polyposis coli protein (APC), and glycogen synthase kinase 3β (GSK-3β). Under normal physiological conditions, this complex can phosphorylate β-catenin, causing the degradation of β-catenin and maintaining its dynamic balance in the cell [9]. Nevertheless, after activating the Wnt signaling pathway, Dvl protein inactivates the above complex, blocking the phosphorylation and degradation of β-catenin, and allowing β-catenin to stably accumulate in the cytoplasm. Subsequently, the stable β-catenin enters the nucleus and binds to T cell factor/lymphoid enhancer factor (TCF/LEF), activating target genes related to cell proliferation, self-renewal, and survival, which is vital for maintaining the stem cell characteristics of GSCs [10]. When the Wnt signal is abnormally activated, a series of adverse biological effects are triggered. The proliferative ability of GSCs is significantly enhanced, the anti-apoptotic mechanism is strengthened, and the self-renewal and differentiation potential is increased. Meanwhile, owing to the activation of the Wnt pathway, the immune evasion mechanism in the tumor microenvironment is also enhanced, inhibiting the recognition and clearance of GSCs by the immune system and further exacerbating the malignancy and treatment resistance of gliomas [11,12]. To deeply understand the mechanism of the Wnt/β-catenin pathway in GSCs, research has concentrated on other key regulators, such as the Dvl (Dishevelled) protein that plays a central role in Wnt signal transduction. This study found that the abnormal activation of Dvl is closely correlated with the high migratory and invasive ability of GSCs, and the research on small molecule inhibitors targeting Dvl indicates that targeting Dvl can significantly lower the invasiveness of GSCs and increase the sensitivity of chemotherapy drugs [13]. It is noteworthy that this pathway also plays a crucial role in non-stem tumor cells, not only by regulating cell cycle progression and anti-apoptotic processes, but also by contributing significantly to invasive tumor growth and immune evasion [14,15]. In addition, GSCs are not a fixed and independent population; tumor cells can dynamically transition between GSC and non-GSC states under the influence of microenvironmental signals [16]. Therefore, blocking Wnt/β-Catenin signaling can inhibit the stemness and drug resistance of GSCs while also exerting anti-proliferative and anti-invasive effects on a broader population of glioma cells, providing more effective control over the overall progression of glioma [17].

### 2.3. Hippo Signaling Pathway

During the physiological operation of normal cells, the Hippo pathway follows a rigorous and orderly regulatory process. Firstly, serine/threonine-protein kinase 1/2 (MST1/2) is activated by specific cellular signal stimuli. This activation event is like turning on the “master switch” for a series of subsequent reactions. The activated MST1/2 acts on large tumor suppressor 1/2 (LATS1/2), aiming to promote its activation [18]. The activated LATS1/2 negatively regulates Yes-associated protein (YAP) and transcriptional co-activator (TAZ) through phosphorylation, resulting in a loss of their biological activity. The inactive YAP/TAZ is retained in the cytoplasm and unable to enter the nucleus to bind to the promoter regions of oncogenes, which, therefore, effectively suppresses the expression of oncogenes, ensuring that the cells are in a normal physiological state and that cellular homeostasis and normal functions can be maintained [19]. However, in glioma cells, the situation is completely opposite. The key regulatory factors MST1/2 and LATS1/2 of the Hippo pathway are often inhibited by various factors and fail to play their regulatory roles normally. This inhibitory state directly causes the abnormal activation of the YAP transcriptional co-activator after it breaks free from normal regulatory constraints [20]. The activated YAP can break through the cytoplasmic limitation and then successfully enter the nucleus. Once in the nucleus, YAP binds tightly to transcriptional enhancer factor-TEF/ATTS DNA-binding domain (TEAD) transcription factors. This binding triggers a series of gene activation processes, mainly involving genes which are correlated with cell proliferation, anti-apoptosis, and stem cell characteristics [21]. The activation of cell proliferation-related genes significantly accelerates the proliferation rate of glioma cells and increases the cell number rapidly; the activation of anti-apoptosis genes enables the cells to escape the normal apoptosis process and survive in an unfavorable environment. Furthermore, the activation of genes related to stem cell characteristics endows tumor cells with stronger self-renewal and differentiation abilities, enabling them to continuously generate new tumor cells and possess stronger adaptability and invasiveness. The combination of these factors contributes to a significant enhancement in the proliferation and invasion abilities of glioma cells, also greatly increasing the resistance to various treatment methods [22]. Eventually, in clinical practice, there is an extremely significant correlation between the overexpression of YAP and the poor prognosis of glioma patients, seriously influencing the treatment effect and the quality of life of patients, posing a huge challenge to the treatment of gliomas [23]. Besides the role of YAP in the Hippo signaling pathway, recent studies have also demonstrated that Src homology 2 domain-containing tyrosine phosphatase 2 (SHP2) plays a vital role in the occurrence and development of gliomas and is associated with the Hippo signaling pathway. Existing research has shown that SHP2 is highly expressed in glioma stem cells (GSCs) and is closely correlated with the expression of SOX2. It is indispensable for maintaining the stem cell characteristics of GSCs. Knocking down SHP2 can significantly inhibit the proliferation and tumorigenicity of GSCs, as verified in the soft agar assay. For example, after knocking down SHP2 in the GSC 83 and GSC 1123 cell lines, colony formation was significantly reduced [24]. Meanwhile, there is a potential association between SHP2 and the Hippo signaling pathway. In addition, research on non-small cell lung cancer has found that the YAP/TAZ/TEAD pathway is related to tumor drug resistance. When SHP2 inhibitors are used in combination with KRAS G12C inhibitors, the therapeutic effect may be enhanced by regulating this pathway [25]. This suggests that in the treatment of gliomas, developing targeted inhibitors against SHP2 or employing a treatment regimen that combines SHP2 inhibitors with other signaling pathway inhibitors and traditional chemotherapeutic drugs, is expected to bring more effective treatment strategies for glioma patients.

### 2.4. Slit/Robo Signaling Pathway

**Figure 1:**
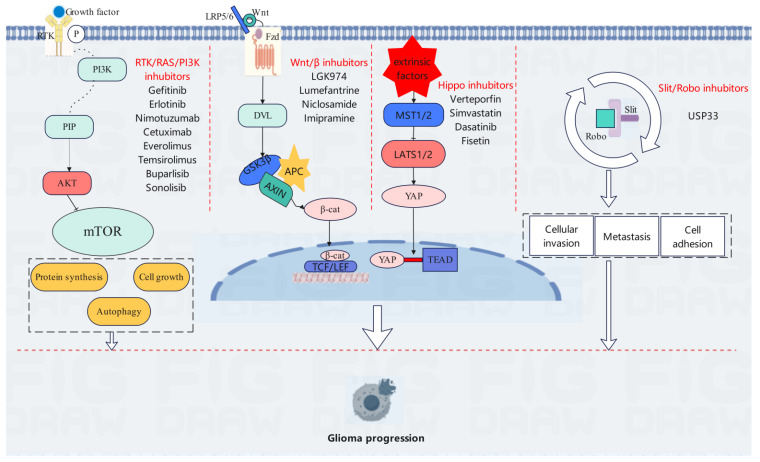
This figure illustrates the signaling pathways related to glioma progression and the role of external factors. Growth factors activate the RTK/RAS/PI3K pathway, regulating protein synthesis, and so on, and there are a variety of inhibitors. Wnt activates the Wnt/β-catenin pathway, stabilizing β-catenin to regulate gene expression, and there are also corresponding inhibitors. In the Hippo pathway, MST1/2 and LATS1/2 usually inhibit YAP. In gliomas, this pathway is often inhibited by external factors, and YAP enters the nucleus and binds to TEAD to promote gene expression. The Slit/Robo pathway is associated with cell invasion and related processes, with its activity being regulated. Abnormalities in these pathways and external factors lead to the deterioration of gliomas.

In the complex pathological process of gliomas, the Slit/Robo signaling pathway is involved in glioma inflammation [26,27,28], cell migration [29], angiogenesis, and immune cell infiltration [30,31], suppressing or promoting the expression of key proteins, including cell adhesion molecules, matrix metalloproteinases, interleukins, angiogenic growth factors, and immune checkpoints [32]. As a vital secreted protein, the Slit protein plays an indispensable role in intercellular communication and regulation. It has a unique molecular structure and can accurately recognize and bind to the Robo receptor. Once the two successfully bind, a series of complex intracellular signal transduction events are triggered, precisely and critically regulating the chemotaxis and mobility of cells. Under normal physiological conditions, this regulatory mechanism contributes to maintaining the orderly migration of cells and the homeostasis of tissues [32]. However, in the special microenvironment of gliomas, the Slit/Robo signaling pathway often falls into a state of abnormal activation or dysregulation. This abnormal state is closely related to complex gene changes and abnormal protein expression in tumor cells as well as the interaction of various cytokines and signaling molecules in the tumor microenvironment [33]. Studies have suggested that in glioma cells, the expression level of Slit may be abnormally increased or decreased, and that the activity and distribution of Robo receptors may change accordingly. These changes break the original delicate signal regulatory balance. When effective means are implemented to inhibit the Slit/Robo pathway, the migration ability of glioma cells is significantly reduced [34]. Based on targeted therapy, it is expected to precisely inhibit the invasiveness of gliomas, providing new hope and more effective treatment options for glioma patients. It will certainly play a critical role in future clinical treatment of gliomas, holding significantly high research value and application prospects [35]. Many signaling pathways, including RTK/RAS/PI3K, Wnt/β-catenin, Hippo/YAP, and Slit/Robo, are crucial for the occurrence and development of gliomas and lay a key molecular foundation for targeted therapy (Figure 1). In recent years, researchers have developed various means, including targeted inhibitors, gene editing tools, and nanocarriers, aiming to inhibit the growth and invasion of gliomas. Next, this study will focus on exploring the application potential of treatment strategies such as targeted inhibitors, gene editing, and nanomedicine targeting these signaling pathways in the treatment of gliomas [36,37,38].

## 3. Targeted Inhibitors

Targeted Inhibitors of the RTK/RAS/PI3K Pathway: The RTK/RAS/PI3K pathway is a vital proliferation-promoting signaling pathway in gliomas. Drugs targeting this pathway include EGFR inhibitors, mTOR inhibitors, and PI3K inhibitors.

EGFR inhibitors: Gefitinib and Erlotinib are two representative EGFR tyrosine kinase inhibitors (TKIs). They effectively prevent the abnormal activation of this pathway by binding to and inhibiting the tyrosine kinase activity of EGFR with high affinity [39,40]. Research has revealed that the cytotoxicity of the anti-EGFR drug gefitinib (10 μM) against the DBTRG.05-MG cell line, which has high EGFR expression, is significantly higher than that against the U87-MG cell line, which has low EGFR expression. In the DBTRG.05-MG cell line, gefitinib also enhances the cytotoxicity of carboplatin and temozolomide, and all the tested drugs can induce apoptosis in a concentration-dependent manner. This indicates that gefitinib has an inhibitory effect on GBM [41]. In a Phase II trial of erlotinib for treating recurrent GBM, patients with recurrence or progression after radiotherapy and chemotherapy were enrolled and received 150 mg of erlotinib daily. An interim analysis of sixteen patients indicated that four had a partial response, one had a partial response in the primary tumor but subsequently developed a new unresponsive lesion, four had stable disease for over 3 months, and seven had tumor progression within 3 months. Although 50% of the patients showed a response, the response was not durable. Although EGFR amplification was present in some tumors, it was not necessarily associated with efficacy. Obviously, erlotinib has a certain inhibitory effect on GBM, while the effect is not durable and has limitations [42]. Antibodies, including Nimotuzumab and Cetuximab, bind specifically to EGFR to block its signal transduction, significantly lowering the proliferative ability of glioma cells. Moreover, Nimotuzumab has played a role in improving the survival period of patients when combined with radiotherapy, providing a novel combination treatment strategy for clinical treatment [43,44].

mTOR inhibitors: Everolimus and Temsirolimus are two mTOR inhibitors. They primarily block the conduction of the R signal by highly specifically inhibiting the activity of mTOR. Everolimus has demonstrated significant antitumor activity in phase II clinical trials and can improve the progression-free survival of some patients with recurrent gliomas, thus offering new hope for treating recurrent gliomas [45,46]. Temsirolimus also reduces the proliferation of glioma cells by blocking mTOR activity, and preclinical studies have also indicated its inhibitory effect on tumor growth, laying a foundation for further clinical research [47].

PI3K inhibitors: Buparlisib (BKM120) is a specific PI3K inhibitor. It blocks the activation of AKT by reducing the production of PIP3, which can therefore effectively decrease cell proliferation and survival [48]. BKM120 has revealed significant efficacy in patients with recurrent gliomas in combination with Temozolomide in clinical trials, providing a novel and effective treatment option for recurrent gliomas [49]. Sonolisib (PX-866) is an analogue of wortmannin and a pan-phosphatidylinositol 3-kinase (PI3K) inhibitor. Its antitumor activity is more potent than that of wortmannin. When used alone or in combination with other drugs, PX-866 exhibits autophagy-promoting, anti-invasion, and anti-angiogenesis effects on GBM cells, and also exerts antitumor impacts on intracranial GBM xenograft mice [50].

Wnt/β-catenin pathway-targeted inhibitors play a crucial role in maintaining GSCs. Targeting this pathway can effectively inhibit the proliferation and survival of GSCs [51].

LGK974: Porcupine is a modifying enzyme of Wnt ligands. LGK974 efficiently inhibits the activity of Porcupine, preventing the secretion of Wnt ligands and consequently significantly reducing the activation of Wnt signals [52]. Studies have suggested that LGK974 effectively inhibits the growth of GSCs in mouse models and exhibits good antitumor effects [53]. A recent study further demonstrated that the combination of LGK974 with chemotherapy drugs can significantly improve the treatment effect of gliomas, especially showing a synergistic effect on inhibiting cancer stem cells, providing a novel strategy for the combination treatment of gliomas [54,55].

Lumefantrine: Lumefantrine is an FDA-approved antimalarial drug. Recent studies have indicated that it can potentially be used for the treatment of GBM. Moreover, it can reverse the resistance of GBM to radiotherapy and temozolomide. Its mechanism of action is to inhibit the Fli-1/HSPB1/epithelial-mesenchymal transition/ECM remodeling protein network. Among them, Fli-1 signaling is associated with the occurrence of GBM and is highly expressed in radiotherapy- and temozolomide-resistant glioblastomas, and can regulate HSPB1 at the transcriptional level. Lumefantrine can bind to Fli-1 and induce apoptosis of parental and resistant GBM cells in vitro. In U87MG glioma cells and resistant GBM orthotopic tumor models, lumefantrine can decrease tumor development in vivo without causing systemic side effects [56].

Niclosamide: This is an FDA-approved anthelmintic drug. Niclosamide can induce cytotoxicity in human GBM cells, accompanied by increased protein ubiquitination, endoplasmic reticulum stress, and autophagy. Niclosamide treatment downregulates survival-promoting signal transduction pathways, including Wnt/β-catenin, PI3K/AKT, MAPK/ERK, and STAT3, reducing the viability of U-87 cells [57]. Recent studies have suggested that the combination of niclosamide and the natural alkaloid camptothecin exerts a synergistic inhibitory effect on the proliferation of the human GBM cell line U87 MG, which may provide a new strategy for treating this disease [58]. The combination of niclosamide and temozolomide can inhibit GBM tumorspheres. Through in vitro treatment of GBM tumorspheres and in vivo mouse model experiments, it has been indicated that the combination can inhibit their cell viability, stemness, and invasiveness, downregulate the expression of related markers, and slow down tumor growth [59,60]. Studies have suggested that the newly synthesized niclosamide-derived compound NSC765689 may target the GBM oncogenic signaling pathway and upregulate miR-135b. Preliminary verification through molecular docking and in vitro screening shows that it can be used as a new treatment drug for GBM [61].

Imipramine: Imipramine is an effective Wnt signaling inhibitor. It induces autophagy and inhibits PI3K/Akt/mTOR. Recent experiments have demonstrated that 60 μM imipramine is cytotoxic and can strongly reduce the colony formation of U87MG and C6 cells, while exerting no impact on primary cultured rat astrocytes and can effectively reduce the invasion of gliomas [62,63,64].

Hippo Signaling Pathway-Targeted Inhibitors: The Hippo signaling pathway is dysregulated in gliomas, which causes the abnormal activation of YAP/TAZ, promoting tumor growth and invasion. Targeting this pathway is beneficial for inhibiting tumor progression.

Verteporfin: Verteporfin is a drug approved for the treatment of macular degeneration. However, in gliomas, it has been found to specifically inhibit the interaction between YAP and TEAD transcription factors, reducing YAP activity and inhibiting the proliferation and migration of glioma cells [65]. In mouse models, Verteporfin has exhibited significant antitumor effects, providing a new drug option for treating gliomas [66].

Simvastatin: This is a commonly used lipid-lowering drug. Glioma research indicates that it reduces YAP activity by activating MST1, inhibiting tumor growth [67]. The combination of Simvastatin and metformin influences the expression of GLUT1 and GLUT6 in glioma cells, inhibits malignant proliferation, and promotes apoptosis, providing novel ideas for the combination treatment of gliomas [68].

Dasatinib: Dasatinib is a multi-target tyrosine kinase inhibitor. It can significantly reduce the migration and invasion ability of glioma cells by inhibiting YAP activity [69]. Through in vitro and in vivo studies and trials in six patients, dasatinib has effectively inhibited the proliferation of glioma cells with various PDGFRA alterations. It demonstrates a synergistic effect with everolimus at low concentrations. Everolimus can also increase the retention of dasatinib in the central nervous system. The combination treatment exhibits good tolerance and provides a new approach for the targeted treatment of these patients [70].

Fisetin: Fisetin is a natural flavonoid compound. It can reduce YAP activity by activating MST1/2 in the Hippo pathway, inhibiting the growth and migration of glioma cells [71]. Studies have found that the co-encapsulation of poorly water-soluble fisetin and cisplatin in liposomes is effective against GBM cells by preparing and characterizing liposomes with different cholesterol/DOPC ratios [72].

Slit/Robo Signaling Pathway-Targeted Inhibitors: The Slit/Robo signaling pathway plays a key role in regulating cell migration and invasion. Therefore, targeting this pathway has been a potential treatment strategy to inhibit the spread of glioma cells. Preliminary studies have indicated significant differences in the expression of Slit2 and Robo1 between healthy brain cells and glioma cells. In control healthy brain cells, the expression of Slit2 reaches the highest level, while its expression is decreased in high-grade gliomas. On this basis, Slit2 is regarded as a tumor suppressor. By contrast, the percentage of glioma cells expressing Robo1 is higher than that of the control group, and Robo1 is identified as an oncogene [73]. However, a recent study has demonstrated that the expression of Slit2 increases with the development of malignancy and is associated with immunosuppression and poor patient survival. The promoting effect of Slit2 on malignant tumors can be caused by the activation of PI3K-γ, further inducing microglia/macrophage chemotaxis and tumor-supportive polarization [74]. In addition, low Slit2 expression is associated with a better prognosis because it can inhibit macrophages, improve tumor vascular function, and enhance sensitivity to chemotherapy and immunotherapy [75]. In vitro studies have suggested that the overexpression of USP33 can significantly inhibit the migratory ability of glioma cells. Mechanistically, USP33 inhibits the migration of glioma cells by regulating the function of the Slit/Robo signaling pathway [76].

Existing studies have shown that inhibitors of signaling pathways, such as RTK/RAS/PI3K, mTOR, Wnt-β-catenin, and Hippo, have exhibited certain efficacy in clinical trials of various solid tumors, including lung cancer and breast cancer. For example, EGFR tyrosine kinase inhibitors (such as gefitinib and erlotinib) can significantly prolong the progression-free survival of patients with EGFR-mutated non-small cell lung cancer (NSCLC), thus becoming one of the first-line treatment options [77]. Wnt or Hippo pathway inhibitors are also gradually attracting attention in early clinical trials of solid tumors, including colorectal cancer and pancreatic cancer. Some studies have shown potential effects in inhibiting tumor proliferation and metastasis [78]. These results indicate that inhibiting the above pathways can bring certain clinical benefits in the treatment of multiple types of tumors, providing ideas for further investigating their applications in the field of gliomas. However, when applied to gliomas (particularly GBM multiforme), the clinical outcomes are not as significant as those in other tumor types. Part of the reason is the highly heterogeneous blood–brain barrier and tumor microenvironment [79]. Obviously, a single targeted therapy often fails to achieve the expected therapeutic effect (Table 1), and the existing clinical trials regarding the above-mentioned pathways are limited (Table 2). Inhibitors targeting different signaling pathways have shown potential in many preclinical and clinical studies. However, the treatment effect may be restricted by tumor drug resistance and microenvironmental factors. Therefore, current research has gradually shifted the focus to the combined application of multiple treatment methods to further improve the treatment effect through synergistic effects. In the following sections, we will deeply explore the combination strategies of targeted inhibitors with other therapies, including immunotherapy, chemotherapy, and radiotherapy, aiming to overcome the complexity and treatment challenges of gliomas.

## 4. Combination Treatment Strategies

Considering the high molecular heterogeneity of gliomas, a single targeted therapy usually fails to achieve the desired treatment outcome. Studies have suggested that the combination of immune checkpoint inhibitors and signaling pathway inhibitors can significantly enhance treatment efficacy. For example, the combined application of PI3K inhibitors and Nivolumab (PD-1) inhibitors can effectively improve the immune response against tumors and significantly decrease the risk of tumor recurrence. In a clinical trial, the combination of Buparlisib and PD-1 demonstrated good clinical results, and the progression-free survival of some patients was prolonged [80]. In addition, the combination of Buparlisib and the anti-angiogenic drug Bevacizumab exhibited significant antitumor effects in mouse models, primarily by reducing angiogenesis and enhancing the immune response to hinder tumor growth [81]. The combination of the MEK inhibitor Cobimetinib and the AKT inhibitor Ipatasertib exhibited significant tumor inhibitory effects in preclinical models, particularly in gliomas with RAS/RAF mutations. This combination therapy acts on multiple targets, not only inhibiting cell proliferation but also reducing the tumor’s resistance to a single drug. Furthermore, recent studies have also indicated that the combination of inhibitors targeting IDH1 mutations, such as Ivosidenib, and PD-1 inhibitors has shown significant efficacy in patients with recurrent gliomas. In a phase II clinical trial, the treatment regimen of Ivosidenib combined with PD-1 resulted in tumor shrinkage in some patients and significantly prolonged progression-free survival [82]. Another successful combination strategy involves the use of the BRAF inhibitor Dabrafenib and the MEK inhibitor Trametinib. This combination has exhibited a better tumor control rate in gliomas with BRAF V600 mutations and has significantly improved the overall survival of patients in clinical trials [83]. However, it should be noted that these combination regimens may also lead to additive immune-related or pathway-specific adverse reactions. For example, common toxicities of immune checkpoint inhibitors include rashes, abnormal liver function, and endocrine disorders [84], while signaling pathway inhibitors (such as PI3K or MEK inhibitors) can cause reactions, including hyperglycemia, fatigue, and diabetic ketoacidosis [85]. In the treatment of glioblastoma multiforme (GBM), the combined inhibition of multiple signaling pathways may enhance the antitumor effect. For instance, the combination therapy of the EGFR inhibitor Erlotinib and the mTOR inhibitor Sirolimus was evaluated in a Phase I clinical trial. The experimental protocol involved using Erlotinib alone for the first 7 days and subsequently adding Sirolimus (a loading dose of 15 mg followed by a maintenance dose of 5 mg) starting on the 8th day. The 3 + 3 classical study design was used for determining the maximum tolerated dose (MTD). The research results demonstrated that this combination could theoretically enhance the antitumor effect [86].

## 5. Immunotherapy

Immunotherapy has exhibited significant potential in the treatment of gliomas, particularly through CAR-T cell therapy and checkpoint inhibitors to improve the immune response against tumors. For example, in a Phase I clinical trial of CAR-T cell therapy targeting EGFRvIII, the tumor burden of some glioma patients was significantly reduced after they received a single peripheral infusion of CAR T-EGFRvIII cells. For example, in a 59-year-old patient with recurrent GBM multiforme, after the treatment, the expression of EGFRvIII in tumor tissue decreased, T-cell infiltration increased with enhanced clonal diversity, early-stage rCBV lowered, and CAR T-EGFRvIII cells persisted in the peripheral circulation for 29 months. Nevertheless, this therapy is still in the clinical trial stage and faces issues such as tumor antigen heterogeneity and immune escape [87]. Nevertheless, owing to the unique immunosuppressive microenvironment of gliomas, the effect of immunotherapy is often limited. As a result, it is essential to combine inhibitors targeting signaling pathways to improve efficacy. The treatment regimen of Pembrolizumab combined with Ipilimumab has also shown potential efficacy in patients with recurrent gliomas. By simultaneously inhibiting the PD-1 and CTLA-4 pathways, T cell activity was enhanced, which could therefore improve the immune response against tumors [88]. In addition, other immunotherapies have also shown promise in research. In a phase I clinical trial, the combination of dendritic cell vaccines and CAR-T cells exerted significant immune activation effects in some patients with recurrent gliomas, with tumor shrinkage and prolonged progression-free survival [89]. In mouse models, this combination strategy also effectively reduced tumor volume. In another study, the combination of TGF-β inhibitors and PD-1 inhibitors effectively reversed the tumor immunosuppressive microenvironment and increased the patient’s treatment response rate [90]. In preclinical studies, TGF-β inhibitors such as Galunisertib combined with PD-1 reduced the tumor’s immune evasion ability by inhibiting the TGF-β signal and simultaneously activated the T cell-mediated antitumor response [91]. This combination therapy significantly enhanced the antitumor effect in animal models, and some patients revealed tumor shrinkage and enhanced immune response in early clinical trials.

## 6. Gene Therapy

As an emerging treatment method, gene therapy also exhibits significant potential in the treatment of gliomas. For example, targeted editing of the EGFRvIII mutation through CRISPR/Cas9 can significantly reduce the proliferation of glioma cells and inhibit tumor growth in animal models [92]. The combination treatment of oncolytic viruses, including Oncorine (H101) and Temozolomide, shows a good synergistic effect, effectively inducing tumor cell lysis and enhancing the chemotherapeutic effect [93]. In addition, recent research has also employed adeno-associated virus (AAV) to deliver the functional PTEN gene into glioma cells to restore the tumor-suppressing function of PTEN [94]. This method significantly reduces tumor growth and enhances sensitivity to Temozolomide. Another gene therapy strategy is to lower the tumor-promoting inflammatory response by silencing the cyclooxygenase-2 (COX-2) gene, thereby inhibiting tumor progression [95]. COX-2 is a key enzyme promoting the inflammatory response in the tumor microenvironment, and its high expression is closely associated with the invasiveness and malignancy of gliomas. Through gene-silencing techniques, including siRNA or shRNA, the expression of COX-2 can be specifically inhibited, which reduces the secretion of pro-inflammatory cytokines, thereby decreasing the proliferation and invasiveness of tumor cells. For example, in a mouse glioma model, silencing the COX-2 gene significantly reduces the tumor growth rate and improves sensitivity to radiotherapy and chemotherapy [96]. These research results demonstrate that gene silencing of COX-2 holds significant clinical potential in inhibiting tumor progression and improving treatment response.

## 7. Application of Nanomedicine in Glioma Treatment

Nanocarriers, which are used as drug delivery tools, can effectively improve the penetration and targeting of drugs. For example, Poly(lactic-co-glycolic acid) nanoparticles carrying bevacizumab can effectively penetrate the blood–brain barrier (BBB), exerting significant tumor-growth-inhibiting effects on glioma mouse models [97]. Doxorubicin carried by silicon nanotubes can increase the drug concentration in tumors and reduce systemic side effects [98]. Liposome carriers such as Doxil (a liposomal doxorubicin) have been employed to improve the BBB penetration of drugs and lower the toxicity of traditional chemotherapeutic drugs [99]. Another type of nanocarrier, called superparamagnetic iron oxide nanoparticles (SPIONs), can significantly increase the accumulation of drugs at the tumor site through magnetic guidance [100]. With nanomedicine technology, drugs can be more precisely delivered to glioma cells, reducing the impact on surrounding healthy tissues and improving the treatment effect. Folic-acid-modified nanoparticles can target glioma cells expressing folate receptors, significantly improving the targeting and antitumor activity of drugs [101]. Moreover, the combination of iron oxide nanoparticles and radiotherapy can not only improve the targeting of drugs but also further enhance the killing effect on gliomas through the magnetothermal effect [102]. Another widely used nano-delivery system is gold nanoparticles modified with polyethylene glycol (PEG). These nanoparticles can target specific tumor cell surface receptors, enhancing the targeting and treatment effect of drugs. Antibody-modified nanocarriers can accurately deliver drugs to tumor cells expressing specific antigens, thus strengthening the selectivity of drugs and reducing side effects [103]. In clinical applications, nanocarrier technology still faces several limitations and challenges. Firstly, there are technical difficulties in the production and large-scale manufacturing of nanoparticles, especially in maintaining their consistency and stability. Furthermore, the in vivo accumulation and biocompatibility of nanoparticles may lead to immune responses or toxicity, consequently restricting their clinical applications. Finally, when applied to GBM, nanocarrier technology still confronts challenges, including biocompatibility, immune rejection, delivery efficiency, and large-scale production. More verification is needed for clinical translation.

## 8. Effects of Irradiation on the Functions of Glioma Cell-Derived Microvesicles and Their Application Potentials

Recently, with the in-depth study of the role of extracellular vesicles (EVs) in tumor biology, the impact of irradiation on glioma cell-derived microvesicles (including exosomes and microvesicles) has gradually become a hot topic of concern. Irradiation not only affects the proliferation and apoptosis of glioma cells themselves, but also alters the distribution and functions of the microvesicles they secrete regarding quantity, protein, and nucleic acid components [104]. Existing studies have indicated that irradiation can promote glioma cells to release more microvesicles carrying DNA repair proteins, immunoregulatory molecules, long non-coding RNAs (lncRNAs), or microRNAs, etc., exerting new regulatory roles in the tumor microenvironment [105]. These microvesicles can serve as vital carriers for extracellular signal transmission, which influences the phenotypic transformation of non-irradiated cells, radiosensitivity, and the immune response process, thereby potentially influencing the overall effect of radiotherapy [106]. In terms of therapeutic applications, glioma cell-derived microvesicles, before and after irradiation, show great potential in drug and nucleic acid delivery, as well as synergistic sensitization. Microvesicles have a lipid bilayer-like structure, low immunogenicity, and good biocompatibility, and can be used as natural carriers for drugs, siRNAs, CRISPR/Cas9 components, etc. [107]. In addition, the carrier properties of microvesicles may change after irradiation, including enhanced tropism for the tumor microenvironment and effects on immune cell activation, etc., providing more plasticity for precise delivery [108]. Modifying or loading these microvesicles using engineering techniques (such as loading chemotherapeutic drugs or gene-editing tools) and combining them with existing radiotherapy and immunotherapy methods is expected to achieve better therapeutic effects, including increasing local drug concentration, reducing systemic side effects, and improving the tumor immune microenvironment.

## 9. The Role of Metabolic Pathways in Glioma Treatment

Glioma cells undergo metabolic reprogramming to adapt to a hostile tumor microenvironment, thereby promoting growth, invasion, and therapeutic resistance [109]. Among these adaptations, glycolysis (the Warburg effect), glutamine metabolism, and lipid metabolism play crucial roles in tumor metabolic plasticity [110]. Even under aerobic conditions, glioma cells depend on glycolysis for energy production and the generation of biosynthetic precursors. Hexokinase 2 (HK2) and pyruvate kinase M2 (PKM2) enhance glycolytic flux, while lactate dehydrogenase A (LDHA) converts pyruvate into lactate, which can thus improve tumor cell survival [111]. Targeting HK2 with 2-deoxyglucose (2-DG) or using LDHA inhibitors can significantly reduce the proliferative capacity of glioma cells [112]. In parallel, gliomas depend heavily on glutamine as a source of carbon and nitrogen. Glutaminase (GLS1) catalyzes glutamine degradation, supporting the TCA cycle and NADPH production, which in turn strengthens antioxidant capacity and sustains cell growth. The GLS1 inhibitor CB-839 effectively suppresses glioma cell growth and improves their sensitivity to radiotherapy and chemotherapy. Cholesterol and fatty acid synthesis are upregulated in glioma cells to maintain cell membrane structure and signal transduction [113]. The key enzymes in these pathways, HMGCR (for cholesterol synthesis) and fatty acid synthase (FASN), are overexpressed in gliomas [114]. Studies have indicated that statins (such as simvastatin) can inhibit HMGCR, thereby suppressing tumor growth, while the FASN inhibitor TVB-2640 has demonstrated promising antitumor activity in preclinical studies [115]. Future studies should integrate metabolomics and personalized treatment strategies to optimize metabolic inhibitors, as well as explore combination regimens with immunotherapy or radiotherapy to enhance therapeutic efficacy and reduce resistance. Recently, the development of metabolic inhibitors has progressed significantly. For example, glycolysis inhibitors, including 2-DG and LDHA inhibitors, have entered clinical trials and shown certain antitumor activity [116]. Furthermore, the GLS1 inhibitor CB-839 has revealed favorable efficacy in clinical studies for multiple solid tumors, including gliomas [117].

## 10. Conclusions and Future Perspectives

The signaling pathways described in this article, such as RTK/RAS/PI3K, Wnt/β-catenin, Hippo, and Slit/Robo, all play vital roles in the occurrence, development, and treatment resistance of gliomas. In-depth research on these pathways has strongly fostered the development of various targeted inhibitors, which have also exhibited certain efficacy in clinical and preclinical studies. Moreover, future research should concentrate more on exploring multi-pathway combined targeted therapy and personalized treatment strategies. For example, combining nanomedicine with emerging technologies, including immunotherapy and gene therapy, may further enhance the antitumor effect. The personalized design of nanocarriers, such as optimizing the targeting of carriers according to specific tumor biomarkers of patients, will also be a vital research direction in the future. In terms of gene therapy, for example, silencing key tumor genes (such as cyclooxygenase-2) through CRISPR/Cas9 gene editing or RNA interference technology can effectively decrease the tumor-promoting inflammatory response and inhibit tumor progression. The application of gene delivery tools (such as adeno-associated virus, AAV vectors) provides novel possibilities for restoring the function of tumor-suppressor genes. Although nanomedicine shows great potential in glioma treatment, its clinical translation still faces many challenges, including biocompatibility, possible immune responses, and difficulties in large-scale production. Furthermore, more clinical studies are needed to verify the safety and effectiveness of these new technologies, which can therefore promote their wide application in clinical treatment.

## Figures and Tables

**Table 1:** Approved drugs targeting the RTK/RAS/PI3K, Wnt/β-catenin inhibitor, Hippo signaling inhibitor, and Slit/Robo signaling inhibitor pathways.

Type		Drug Name	Suggested Mechanism of Action	References
RTK/RAS/PI3K Inhibitors	EGFR Inhibitors	Gefitinib	Binding to and inhibiting the tyrosine kinase activity of EGFR, preventing abnormal activation of the pathway.	[33,34]
		Erlotinib		[39,40]
		Nimotuzumab	Binding to EGFR and blocking its signal transduction.	[43,44,45]
		Cetuximab		[43,44,45]
	mTOR Inhibitors	Everolimus	Inhibiting mTOR activity and blocking PI3K/AKT/mTOR signal transduction.	[45,46]
		Temsirolimus		[47]
	PI3K Inhibitors	Buparlisib	Reducing PIP3 production, blocking AKT activation, and decreasing cell proliferation and survival.	[48,49]
		Sonolisib	Inhibiting the activity of pan-phosphatidylinositol 3-kinase (PI3K) and exerting effects, including promoting autophagy, anti-invasion, and anti-angiogenesis.	[50]
Wnt/β Inhibitors	Porcupine Inhibitors	LGK974	Inhibiting the activity of Porcupine, preventing the secretion of Wnt ligands, and reducing Wnt signal activation.	[52,53,54]
	Antimalarial Drug	Lumefantrine	Inhibiting the Fli-1/HSPB1/epithelial-mesenchymal transition/ECM remodeling protein network and reversing drug resistance.	[56]
	Anthelmintic Drug	Niclosamide	Inducing cytotoxicity in human GBM cells and down- regulating the pro-survival signal transduction pathway.	[57,58,59,60,61]
	Antidepressant	Imipramine	Inducing autophagy and inhibiting PI3K/Akt/mTOR.	[62,63,64]
Hippo Inhibitors	For Macular Degeneration	Verteporfin	Inhibiting the interaction between YAP and TEAD transcription factors and reducing YAP activity.	[65,66]
	Lipid—lowering Drug	Simvastatin	Activating MST1 to reduce YAP activity.	[67,68]
	Tyrosine Kinase Inhibitor	Dasatinib	Inhibiting YAP activity and reducing migration and invasion ability.	[69,70]
	Flavonoid	Fisetin	Activating MST1/2 in the Hippo pathway to reduce YAP activity.	[71,72]
Slit/Robo Inhibitors	Deubiquitinase	USP33	Regulating the function of the Slit/Robo signaling pathway and inhibiting glioma cell migration.	[76]

**Table 2:** Ongoing clinical trials for adult gliomas (Data sourced from ClinicalTrials.gov, accessed on March 10, 2025).

Pathway/Classification	Drug Name	Type of Glioma (Indication)	Adjuvant/Combination Therapy	Clinical Trial ID (Phase)	Sponsors	Expected Enrollment
RTK/RAS/PI3K	Everolimus	Newly diagnosed high-grade glioma (HGG), including DIPG	Ribociclib	NCT05843253 (Phase II)	Nationwide Children’s Hospital	100 (estimated)
	Afatinib/Dasatinib/Everolimus	Recurrent glioblastoma	Palbociclib/Olaparib	NCT05432518 (Early Phase 1)	AHS Cancer Control Alberta	10
	Paxalisib	Diffuse midline gliomas (including DIPG)	ONC201, Panobinostat	NCT05009992 (Phase 2)	University of California, San Francisco	360
	AMG386/Bevacizumab	Recurrent glioblastoma (GBM)	—	NCT01290263 (Phase I/II)	Dana-Farber Cancer Institute	48
Wnt/β-catenin	Genetic Testing-based Treatment	Glioblastoma (including various solid tumors)	None (diagnostic study)	NCT05432518	Hospices Civils de Lyon	410 (estimated)

## Data Availability

No new data were created for this review. The content of this article is based on a comprehensive analysis of the published literature.
